# Reporting Animal Studies: Good Science and a Duty of Care

**DOI:** 10.1371/journal.pbio.1000413

**Published:** 2010-06-29

**Authors:** Catriona J. MacCallum

**Affiliations:** Senior Editor, PLoS Biology, Public Library of Science, Cambridge, United Kingdom

Estimates of the number of non-human animals used in research vary between 10 million and 50 million [1],[2]. As a publisher, we receive submissions from scores of research groups in numerous different countries that report experimental results or observational data about a huge variety of organisms. Each field, each location, each type of experiment, and each organism may carry a different set of standards governing how the research is approved at an institutional or national level and how the study is reported. Despite this eclectic mix of guidelines and standards, many species used in research are not regulated in any formal manner.

What role, therefore, should editors or publishers play in defining and policing the standards of reporting on animal studies in their journal? Like many journals, *PLoS Biology's* current policy (and that for all the PLoS journals) states that:

“*All animal work must have been conducted according to relevant national and international guidelines. In accordance with the recommendations of the Weatherall report, The use of non-human primates in research, we specifically require authors to include details of animal welfare and steps taken to ameliorate suffering in all work involving non-human primates*.”

Is this enough? Not according to a survey [3] of the editorial policies of 288 English-speaking peer-reviewed science journals conducted by the Royal Society for the Prevention of Cruelty to Animals (RSPCA), a UK-based animal welfare organisation. Simply requiring compliance with relevant guidelines and encouraging authors to conform to appropriate welfare standards would earn journals a score of two out of a maximum 12 points for ensuring humane animal research (as estimated in a *PLoS Medicine* “Speaking of Medicine” blog [4]). PLoS journals fare better than most, however; the RSPCA report showed that 50% of the journals responding to their survey (125/236) had either no editorial policy or no meaningful policy relating to animal use in the research they published.

Editors and publishers have two equally important responsibilities with regard to any animal study. The first concerns the ethical treatment of animals (for example, compliance with “the 3Rs” [5]), as highlighted by the RSPCA survey [3], and the second concerns sound science, meaning that any published study ought to be of sufficient scientific quality to ensure that the conclusions are validated and that the work can be replicated and built upon appropriately. They are interlinked, of course, because if animals are to be used in research, then they should not be “wasted” or made to suffer needlessly—work on animals should count.

A more recent survey, published by Kilkenny et al. in *PLoS ONE* [6], emphasises the extent to which we should be concerned about the scientific quality of papers. In their analysis of 271 articles in Medline and EMBASE reporting research on rats, mice, and non-human primates, the authors found that studies often contained no hint of a hypothesis, no randomisation, inappropriate controls, statistical tests without any explanation, no mention of the sex or age of the animal involved, and so on. In other words, they contained a catalogue of basic and fundamental errors that you would not expect in any properly constructed paper from a practicing scientist. Independent of any ethical issue, the consequences of such inappropriate design and inadequate reporting can have profound scientific consequences. Most preclinical laboratory experiments reveal a sex bias in choosing animals—males tend to be easier and cheaper to house and maintain—but focusing on one sex means that important sex differences are overlooked [7],[8]. Moreover, badly reported or unreported studies potentially diminish the extent to which animal models of disease can reliably inform us about clinical interventions to treat the disease in question. In a recent *PLoS Medicine* article, van der Worp et al. [9] explain how such “failed translation” can arise, for example, when only positive results are published and these data are aggregated and included in meta-analyses, systematic reviews, or other synthetic analyses. The impact of such publication bias was made clear in a related *PLoS Biology* article, which analyzed published papers on the interventions tested in animal studies of acute ischaemic stroke. Sena et al. [10] estimate that about 16% of animal experiments, potentially involving 3,600 animals, were not reported. Had the experiments been included, the efficacy of the drug being tested would have dropped from 31.3% to about 23.8%—that is, by about a third. As the authors note, “It seems highly unlikely that the animal stroke literature is uniquely susceptible to the factors that drive publication bias.” Encouraging researchers to publish their negative or neutral results is one way to prevent such bias (and there are open-access venues, such as *PLoS ONE*, to enable this [11]). But, van der Worp et al. suggest, the only way to enforce this would be to have a central register of animal experiments performed—equivalent to clinical trial registration systems—and ensure that registration is referenced in publications.

It is simply unethical to fail to report or to report badly the results of any animal study. We therefore welcome and strongly endorse the initiative of Kilkenny and colleagues, published in this issue of *PLoS Biology*, that outlines a set of author guidelines for reporting on animal studies [12]. The guidelines include a checklist of 20 items “describing the minimum information that all scientific publications reporting research using animals should include,” incorporating such basic parameters as the sex and number of animals in the study and details of their health status and husbandry. The authors' aim is to promote high quality, comprehensive reporting, and ensure an accurate critical review of what was done and what was found.

The guidelines have been developed in consultation with the wider scientific community, journal editors, and major UK bioscience research funding bodies, including the Medical Research Council and the Wellcome Trust. These guidelines apply not only to experimental studies involving, for example, preclinical testing, but also to all bioscience research using animals, from cell biology to behavioural ecology. Kilkenny et al. [12] outline how these guidelines were developed and the rationale behind them. They will be the first guidelines for reporting animal research to be included in the instructions for authors of selected bioscience journals, including those published by Wiley-Blackwell and Springer, as well as PLoS.

All the PLoS journals will recommend these guidelines to their authors. As Kilkenny et al. note in their Perspective article, “The guidelines are not intended to be mandatory or absolutely prescriptive, nor to standardise or formalise the structure of reporting. Rather, they provide a checklist that can be used to guide authors preparing manuscripts for publication, and by those involved in peer review for quality assurance, to ensure completeness and transparency”[12].

This will not be the last word. There are parallel initiatives occurring elsewhere, including, for example, an ongoing project organised by the National Academy of Sciences [13], sponsored by the US Department of Health and Human Services, which is due to report in October this year. The stated aim of the project is to outline the information regarding animal studies that should be included in scientific papers to ensure that the study can be replicated. There is also a conference to be held in August in Washington, D.C., organised by the Physicians Committee for Responsible Medicine among others, to discuss the scientific and ethical imperatives associated with animal research [14]. We will update our guidelines as appropriate when these and related projects deliver updated recommendations.

Publishers, funders, editors, authors, and readers all have a responsibility to ensure the best possible reporting of animal studies. We hope that by publishing these guidelines and through the coordinated efforts of other journals and organisations we will uphold a better duty of care to the animals used in research than we have done to date, and ensure that these studies add meaningfully to the scientific record. Although we will not yet mandate that all the relevant information be included in every published study, it is likely that we will revisit this issue over the coming months once response to the guidelines has been received. Two of the questions we need to address are whether we should mandate such guidelines and how we should then enforce such a policy (for example, without adding unduly to the burden of editors and reviewers). We welcome your comments and feedback about these issues in general and the guidelines in particular.
